# Supplementary figures and images for: STAT3 is a genetic modifier of TGF-beta induced EMT in KRAS mutant pancreatic cancer
Source: eLife. 2024 Apr 4;13:RP92559. doi: 10.7554/eLife.92559 (PMC10994661; doi:10.7554/eLife.92559)

## Original Western Blot Images

Fig 1A

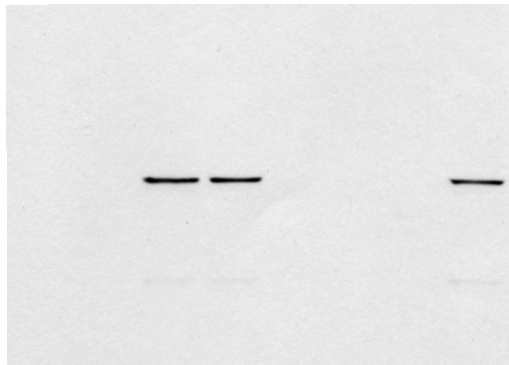

Fig 1A

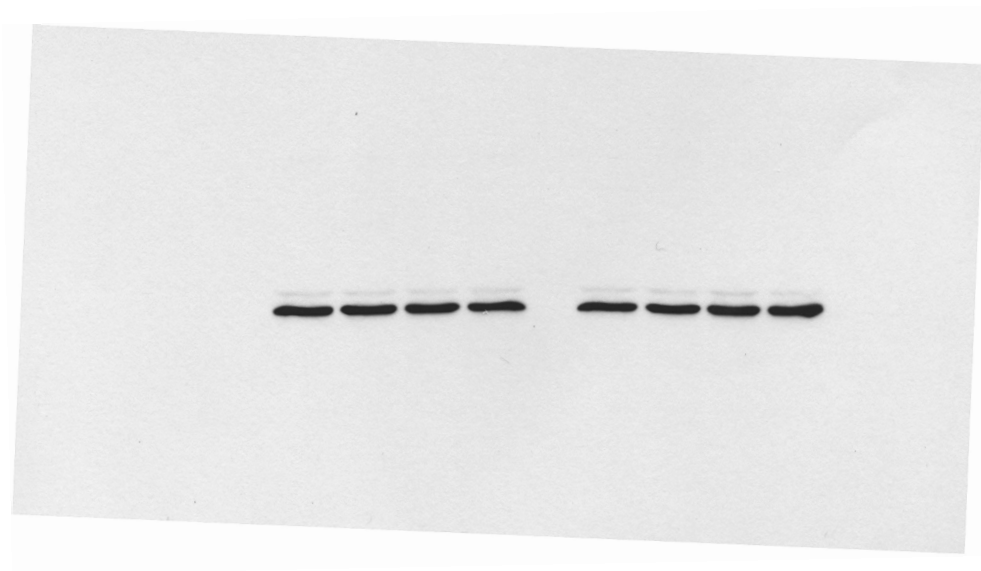

Supplement: Figure 1—source data 1. [file elife-92559-fig1-data1.zip › Figure 1/Figure 1-source data 1.pdf]

Original Western Blot Images

Fig 1A

STAT3 >

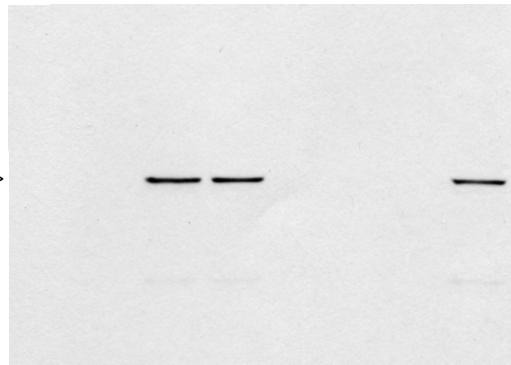

Fig 1A

ERK1/2 >

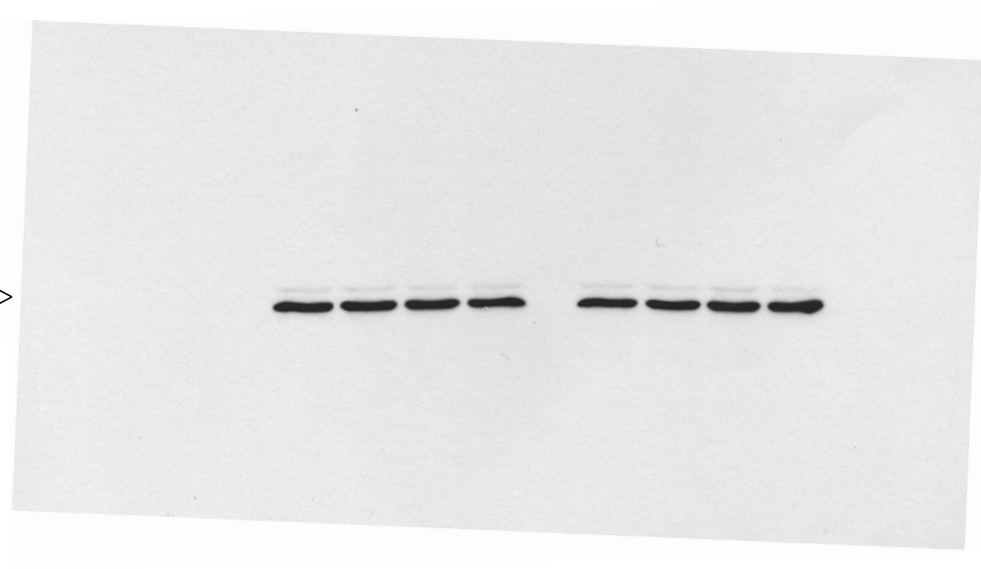

Supplement: Figure 1—source data 1. [file elife-92559-fig1-data1.zip › Figure 1/Figure 1-source data 2.pdf]

## Original Western Blot Images

Fig 1B

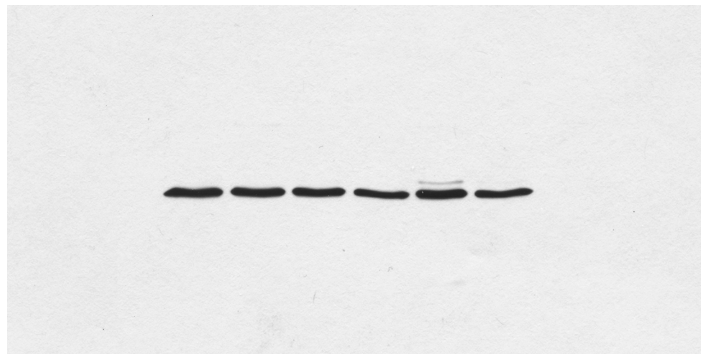

Fig 1B

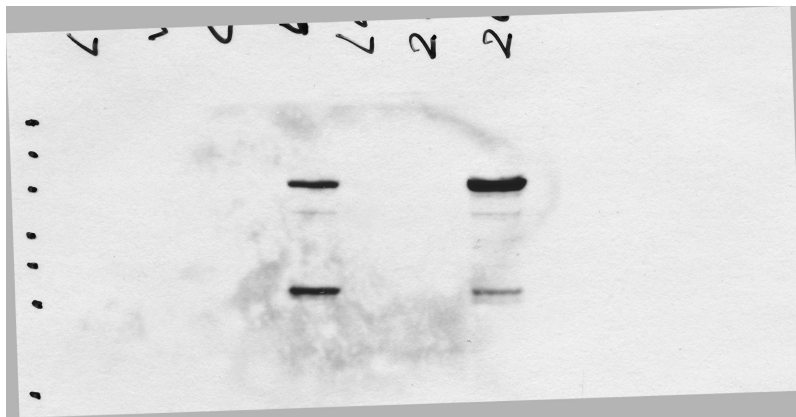

Fig 1B

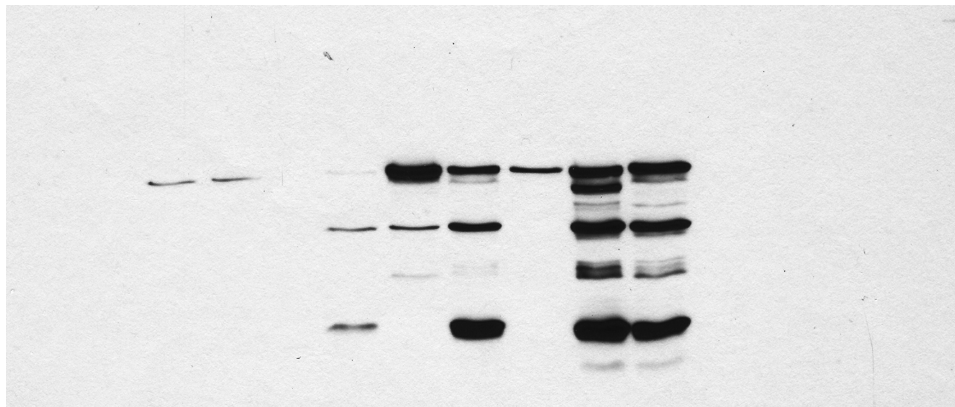

Supplement: Figure 1—source data 1. [file elife-92559-fig1-data1.zip › Figure 1/Figure 1- source data 3.pdf]

Original Western Blot Images

Fig 1B

ERK1/2 >

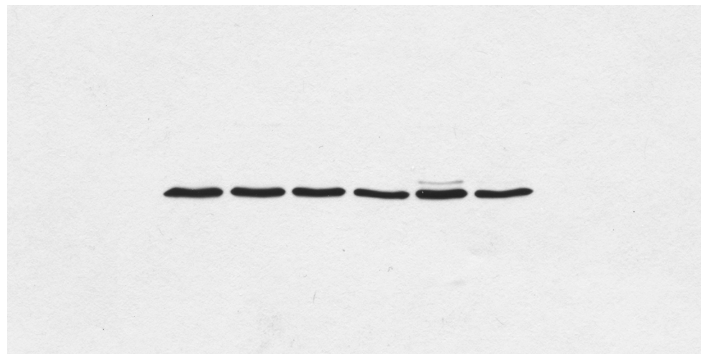

Fig 1B

Phospho-STAT3 >

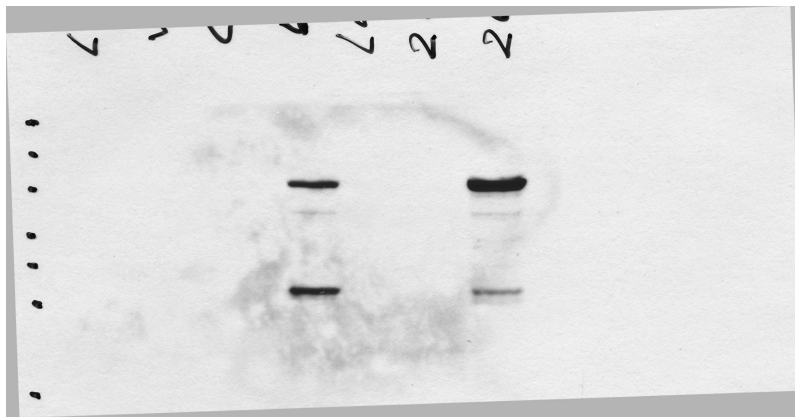

Fig 1B

STAT3 >

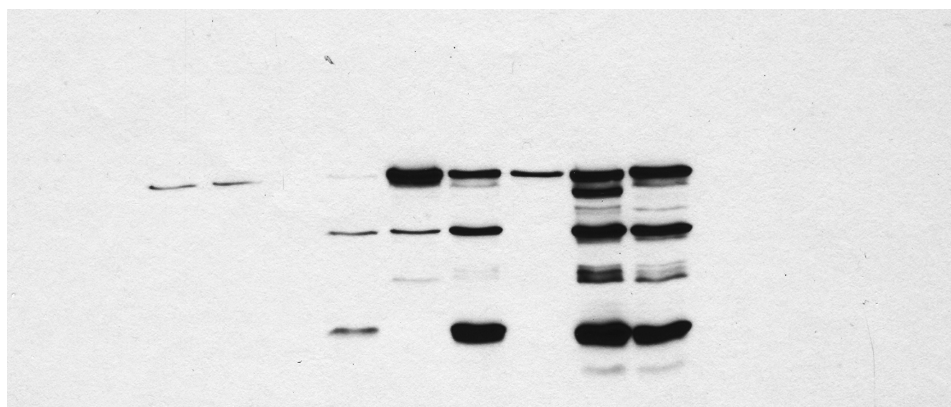

Supplement: Figure 1—source data 1. [file elife-92559-fig1-data1.zip › Figure 1/Figure 1- source data 4.pdf]

## Original Western Blot Images

Suppl Fig. 1B

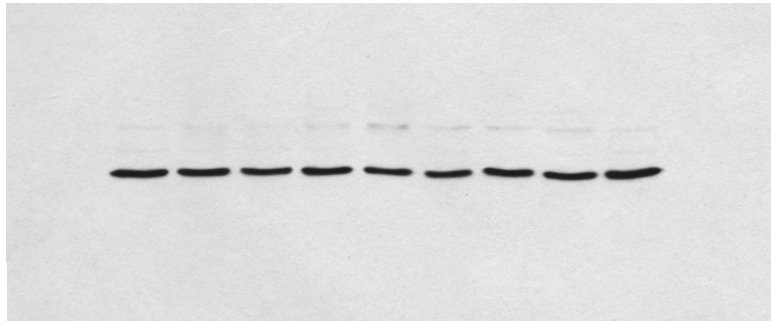

Suppl Fig. 1B

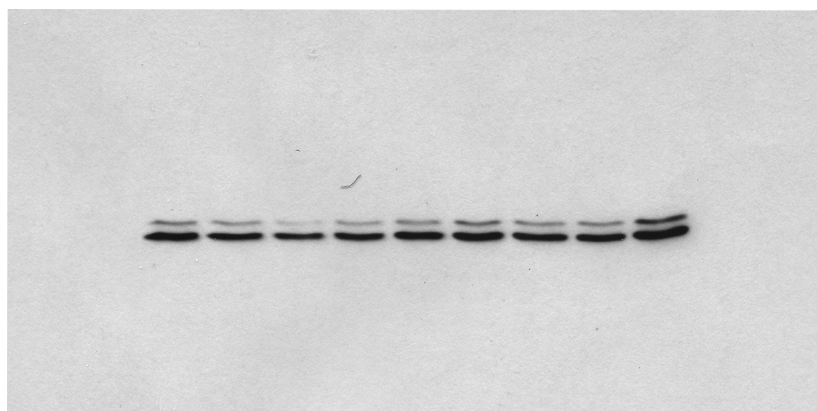

Suppl Fig 1B

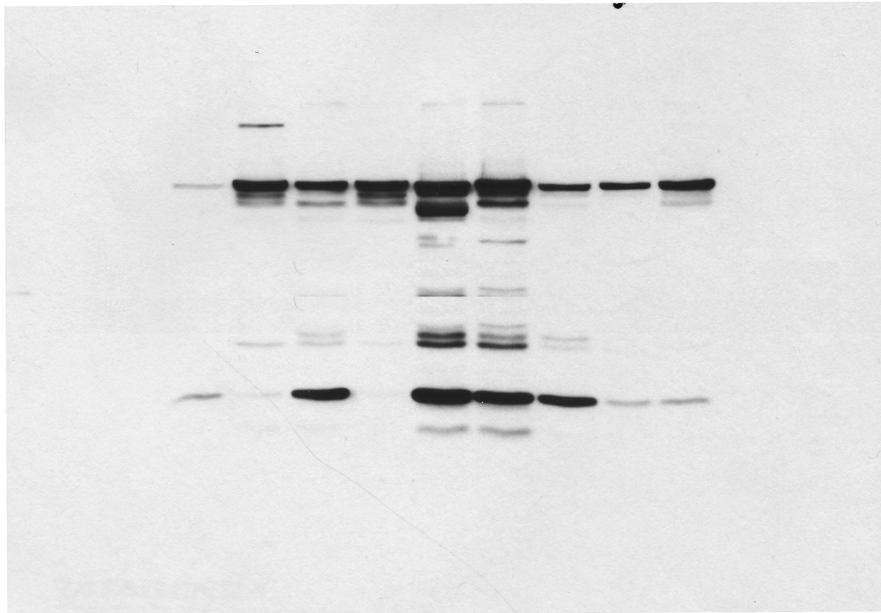

Suppl Fig 1B

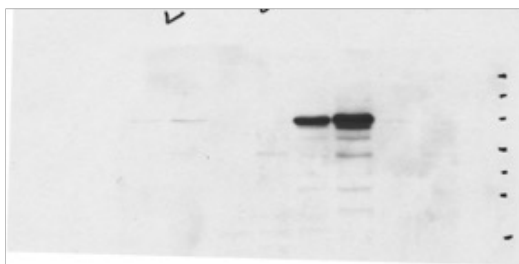

Suppl Fig 1B

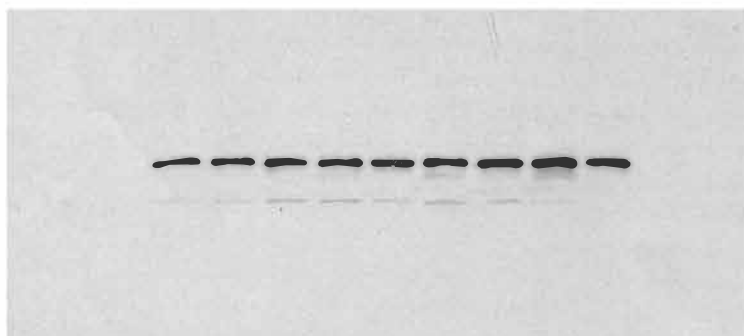

Supplement: Figure 1—figure supplement 1—source data 1. [file elife-92559-fig1-figsupp1-data1.zip › Suppl Figure 1-source data/Suppl Figure 1-source data 1.pdf]

## Original Western Blot Images

Suppl. Figure 1E

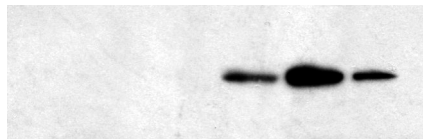

Suppl. Figure 1E

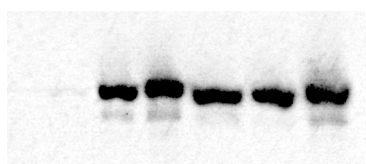

Suppl. Figure 1E

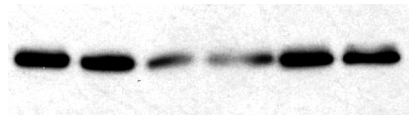

Supplement: Figure 1—figure supplement 1—source data 1. [file elife-92559-fig1-figsupp1-data1.zip › Suppl Figure 1-source data/Suppl Figure 1-source data 3.pdf]

## Original Wester Blot Images

Suppl. Figure 1H

SMAD4 >

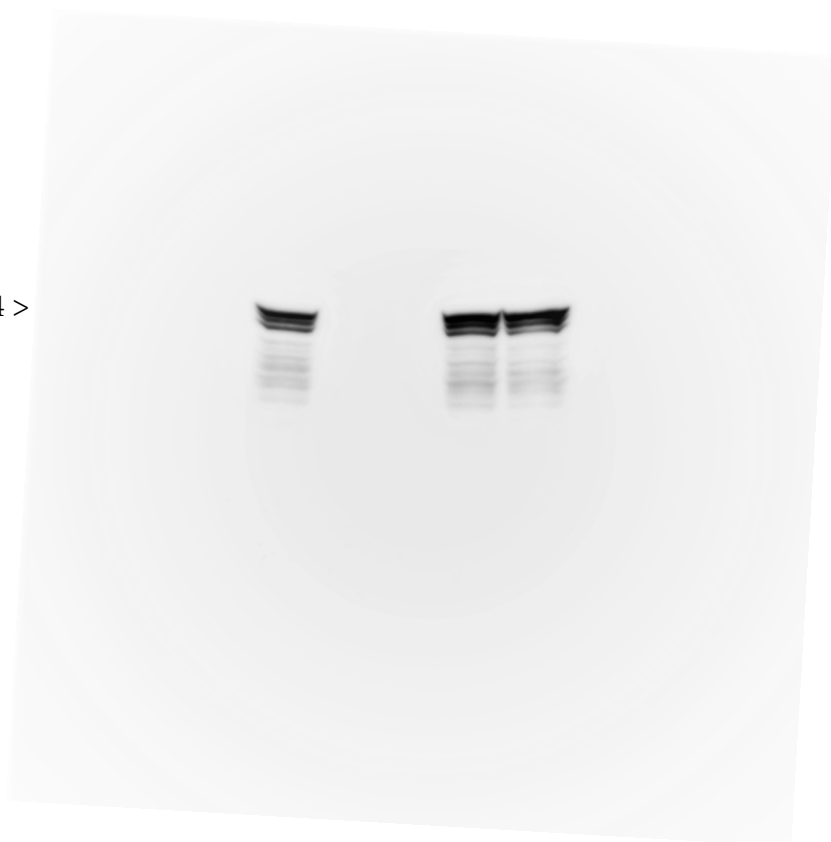

Suppl Figure 1H

TGFBR2 >

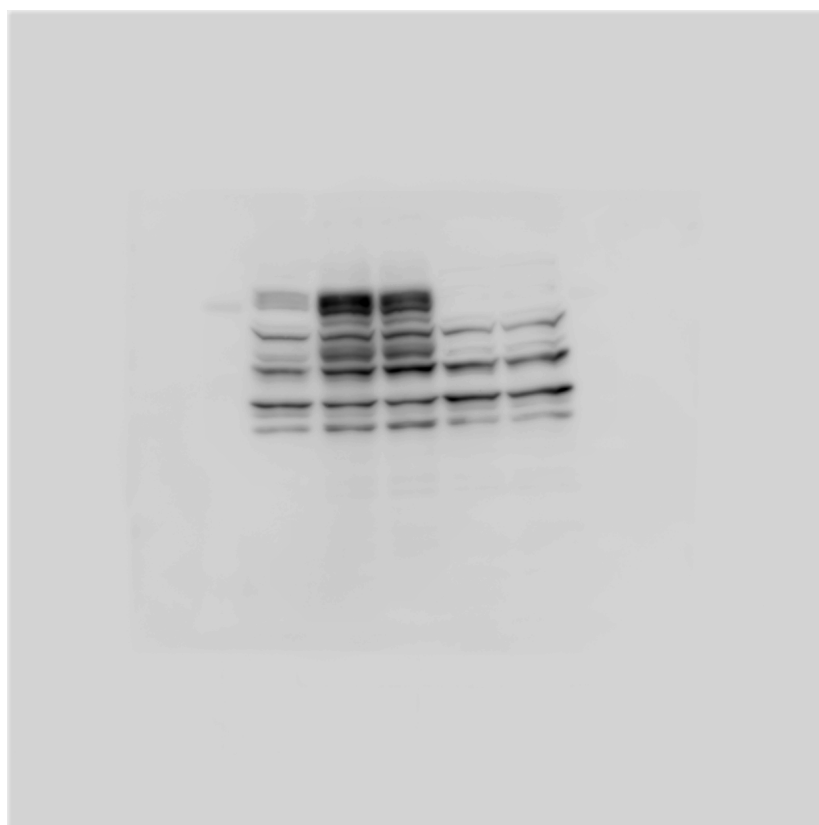

Suppl Figure 1H

ERK1/2 >

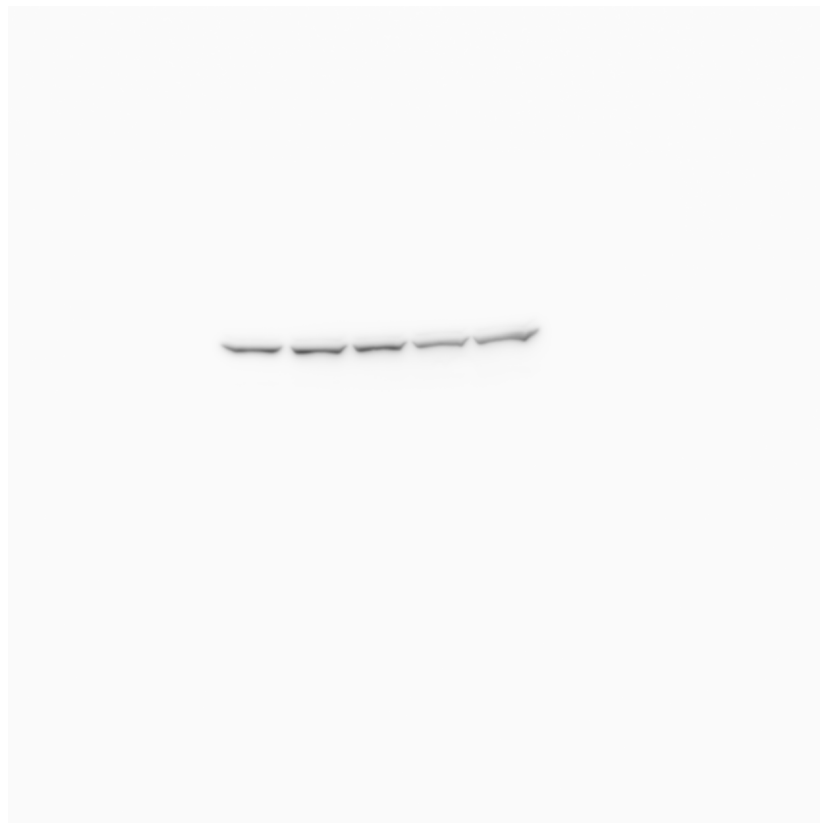

Supplement: Figure 1—figure supplement 1—source data 1. [file elife-92559-fig1-figsupp1-data1.zip › Suppl Figure 1-source data/Suppl Figure 1-source data 6.pdf]

Original Western Blot Images

Suppl. Figure 1E

Phospho-STAT3 >

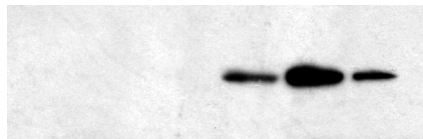

Suppl. Figure 1E

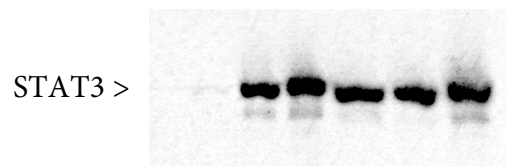

Suppl. Figure 1E

ERK1/2 >

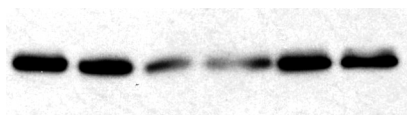

Supplement: Figure 1—figure supplement 1—source data 1. [file elife-92559-fig1-figsupp1-data1.zip › Suppl Figure 1-source data/Suppl Figure 1-source data 4.pdf]

## Original Wester Blot Images

Suppl. Figure 1H

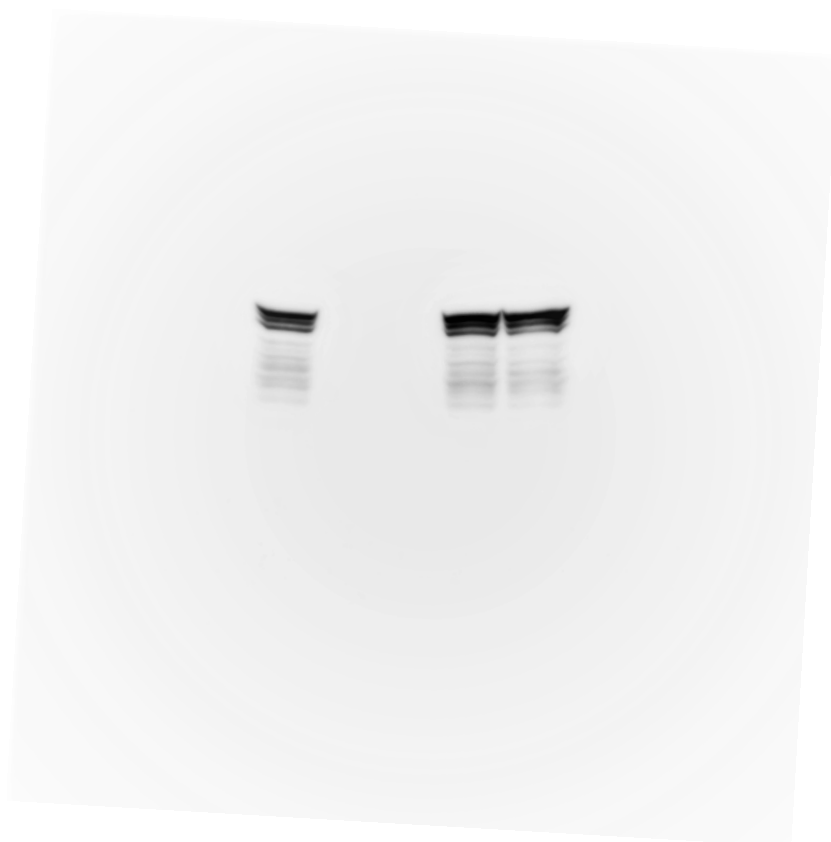

Suppl Figure 1H

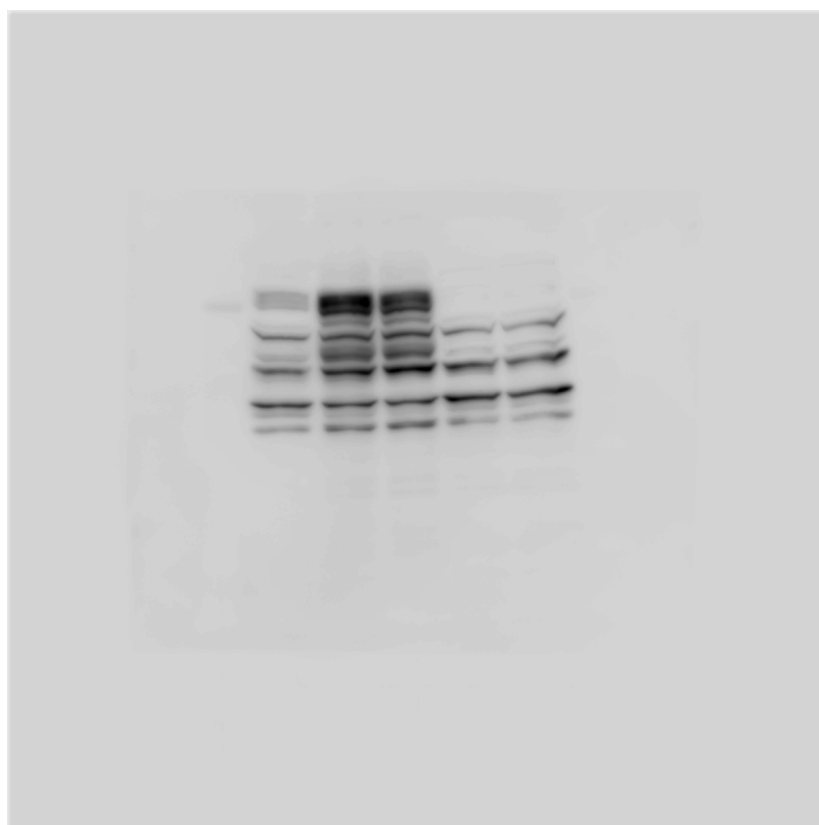

Suppl Figure 1H

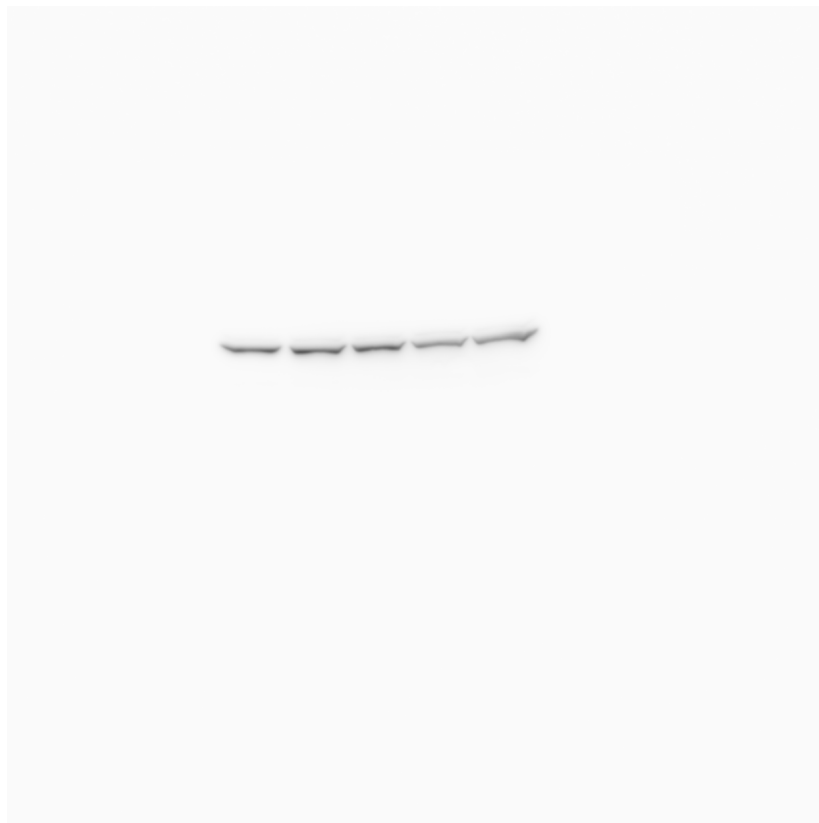

Supplement: Figure 1—figure supplement 1—source data 1. [file elife-92559-fig1-figsupp1-data1.zip › Suppl Figure 1-source data/Suppl Figure 1-source data 5.pdf]

## Original Western Blot Images

Fig 2B

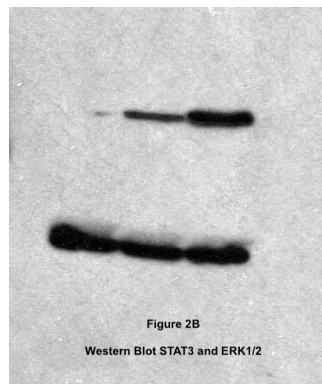

Supplement: Figure 2—source data 1. [file elife-92559-fig2-data1.zip › Figure 2/Figure 2-source data 1.pdf]

Original Western Blot Images

Fig 2B P-STAT3 and ERK

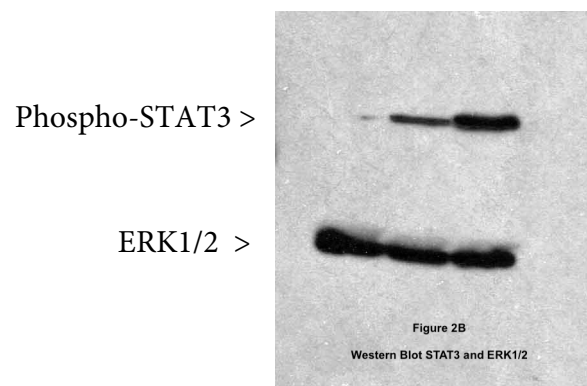

Supplement: Figure 2—source data 1. [file elife-92559-fig2-data1.zip › Figure 2/Figure 2-source data 2.pdf]

Original Western Blot Image

Suppl Fig 2A

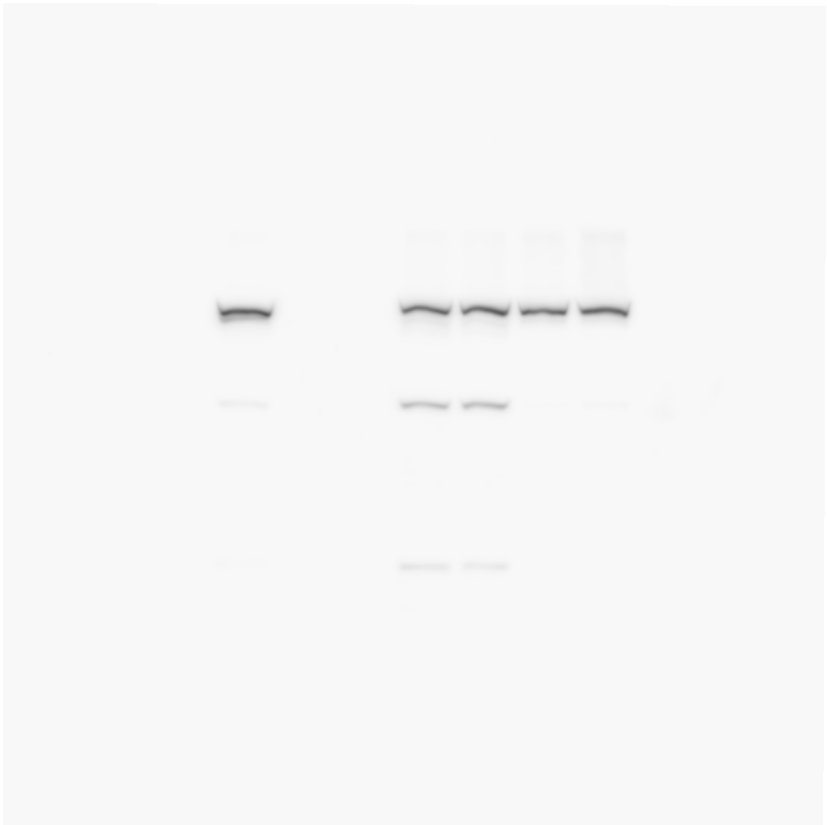

Suppl Fig 2A

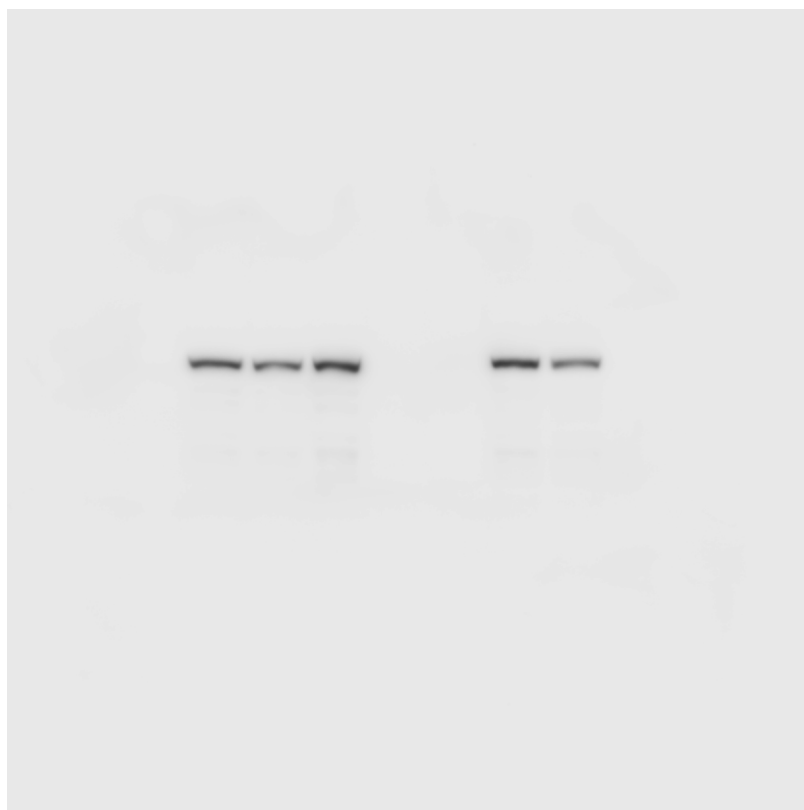

Suppl Fig 2A

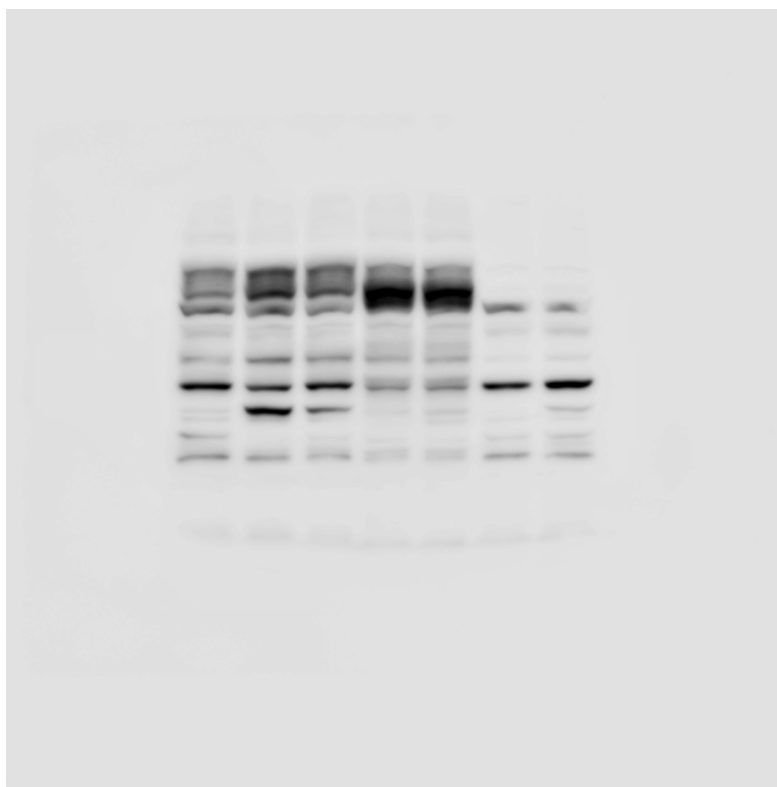

Suppl Fig. 2A

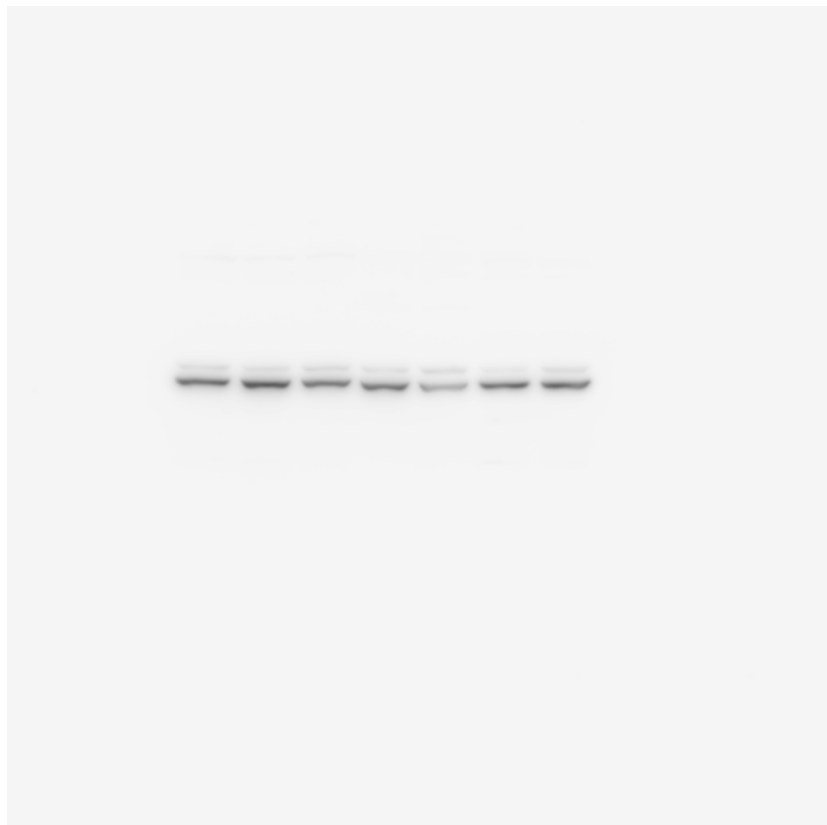

Supplement: Figure 2—figure supplement 1—source data 1. [file elife-92559-fig2-figsupp1-data1.zip › Suppl Figure 2-source data/Suppl Figure 2-source data 1.pdf]

Original Western Blot Image

Suppl Fig 2A

STAT3 >

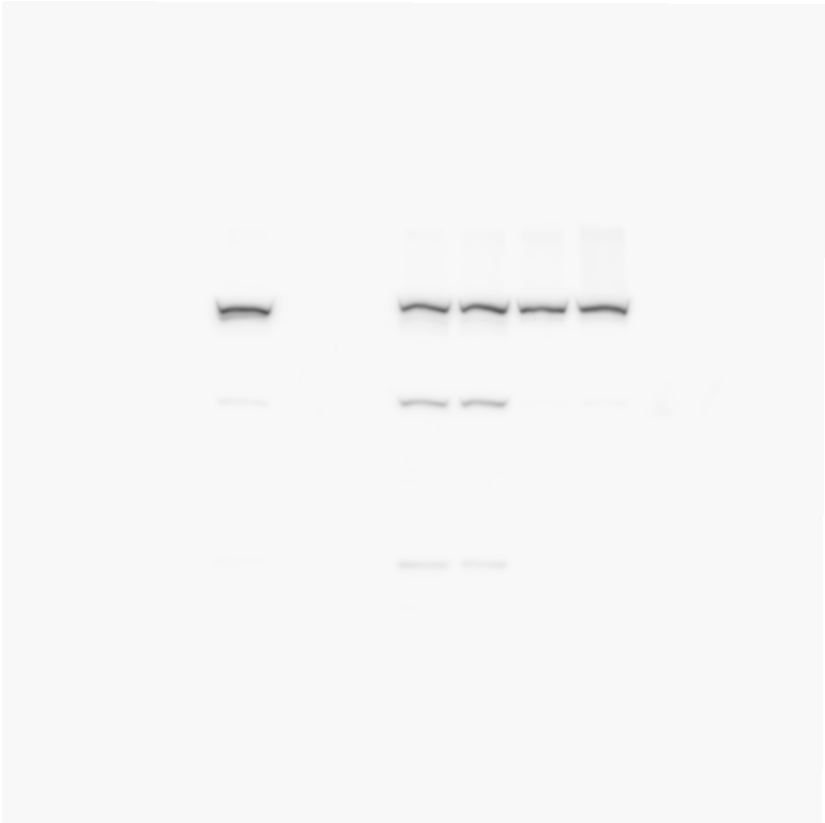

Suppl Fig 2A

SMAD4 >

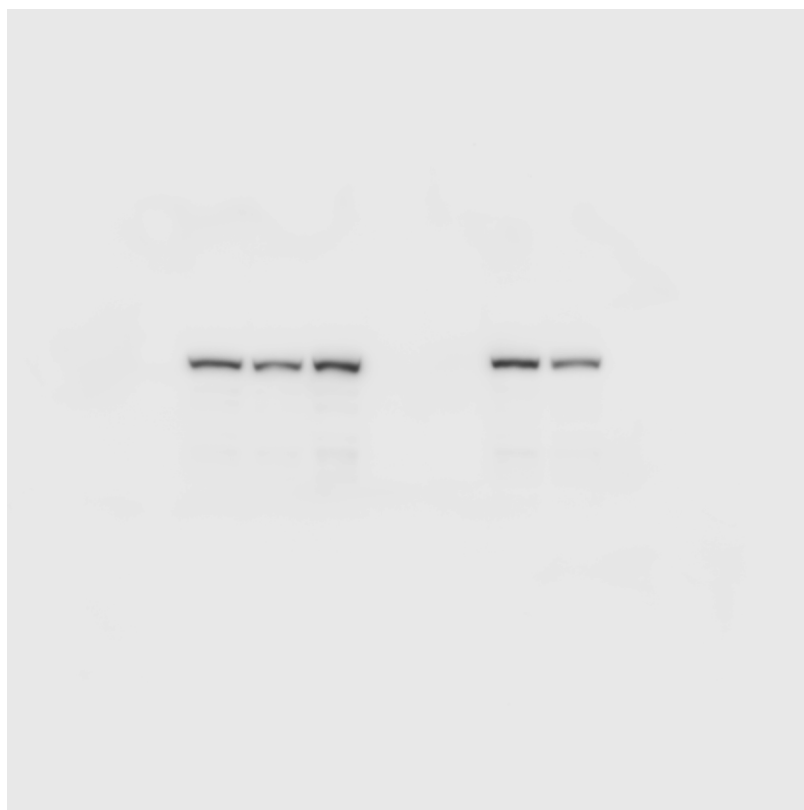

Suppl Fig 2A

TGFBR2 >

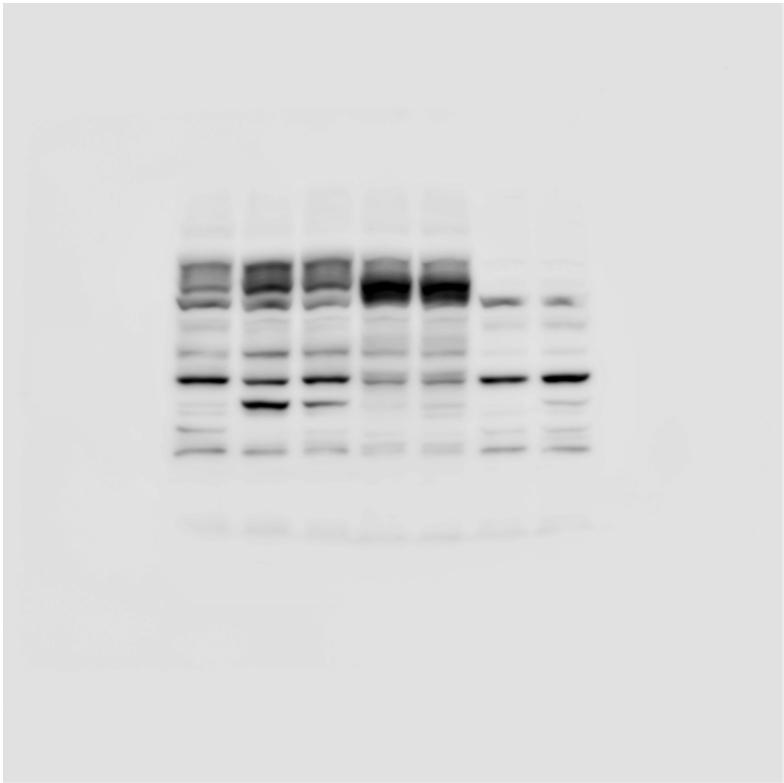

Suppl Fig. 2A

ERK1/2 >

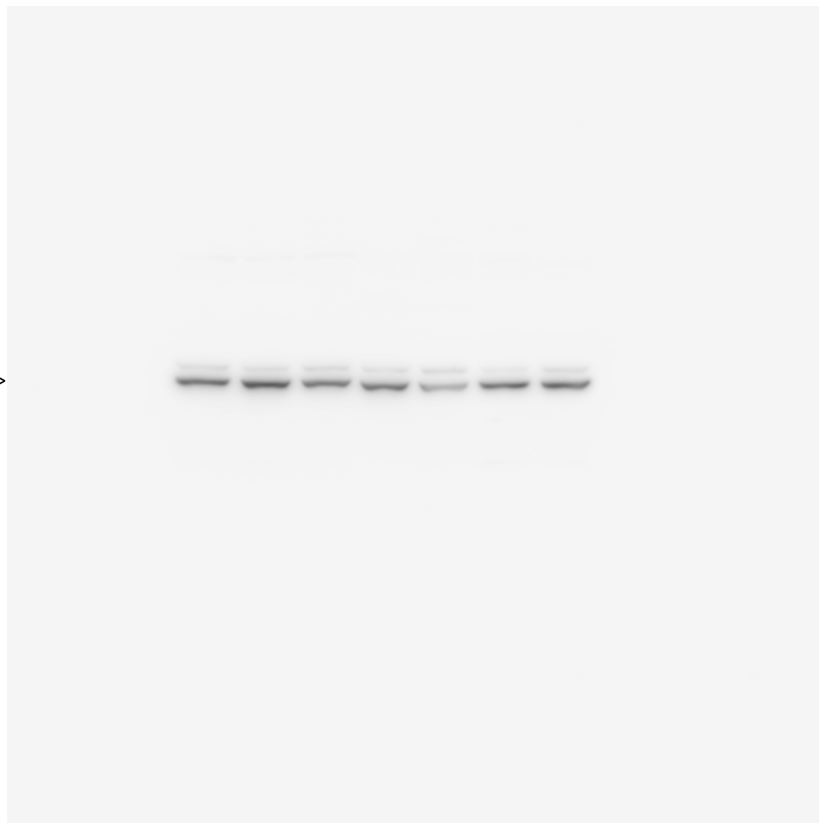

Supplement: Figure 2—figure supplement 1—source data 1. [file elife-92559-fig2-figsupp1-data1.zip › Suppl Figure 2-source data/Suppl Figure 2-source data 2.pdf]
