# Supplementary material for: STAT3 is a genetic modifier of TGF-beta induced EMT in KRAS mutant pancreatic cancer
Source: eLife. 2024 Apr 4;13:RP92559. doi: 10.7554/eLife.92559 (PMC10994661; doi:10.7554/eLife.92559)
Supplement: Figure 1—figure supplement 1—source data 1. [file elife-92559-fig1-figsupp1-data1.zip › Suppl Figure 1-source data/Suppl Figure 1-source data 2.pdf]

Original Western Blot Images

Suppl Fig. 1B

ERFK1/2 >

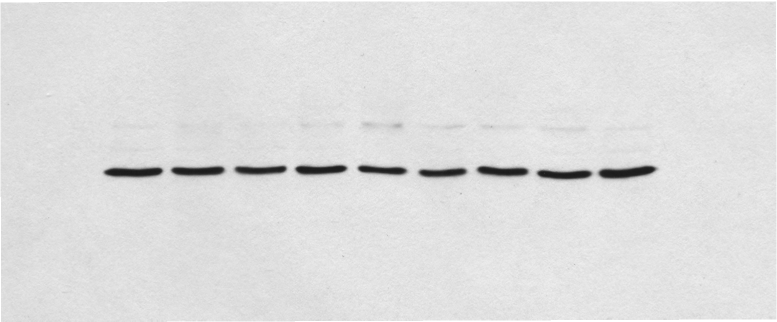

Suppl Fig. 1B

Phospho-ERK >

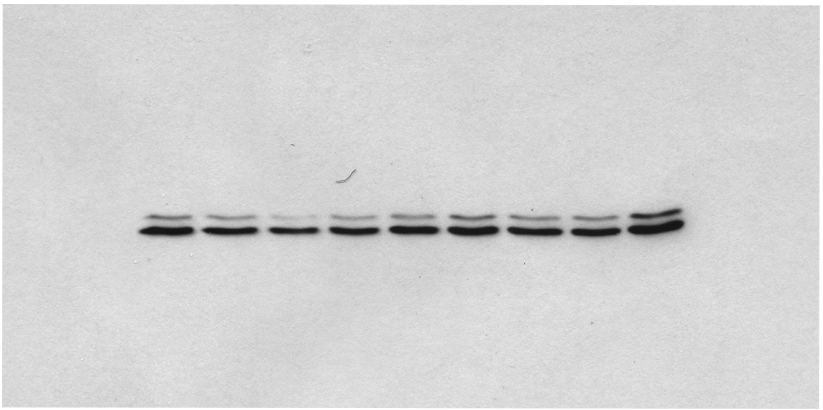

Suppl Fig 1B

STAT3 >

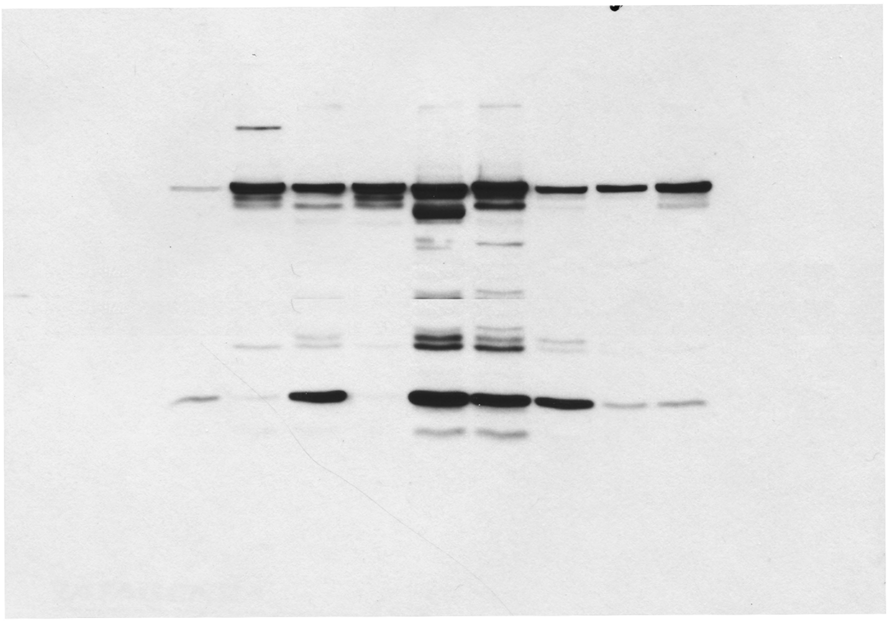

Suppl Fig 1B

Phospho-STAT3 >

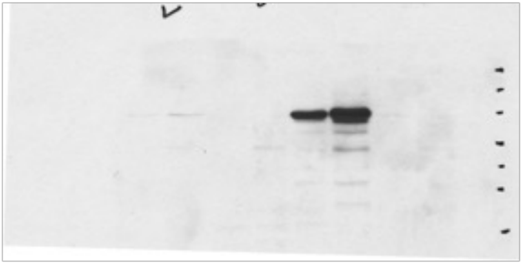

Suppl Fig 1B

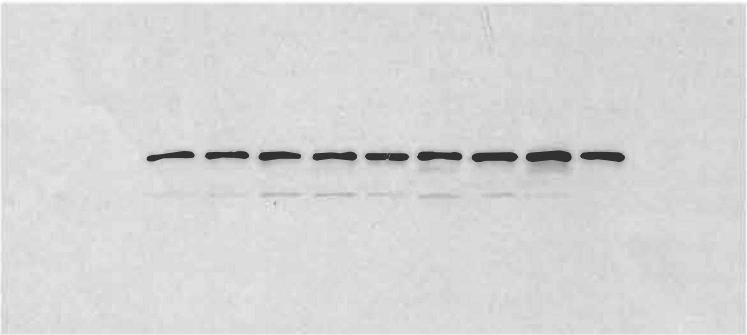

AKT >
