# Supplementary material for: STAT3 is a genetic modifier of TGF-beta induced EMT in KRAS mutant pancreatic cancer
Source: eLife. 2024 Apr 4;13:RP92559. doi: 10.7554/eLife.92559 (PMC10994661; doi:10.7554/eLife.92559)
Supplement: Figure 4—source data 2. — Gene signatures were derived from Tan et al., 2014;6 (10):1279–93. [file elife-92559-fig4-data2.docx]

**Figure 4 -source data 2:** Signature genes used to designate human PDAC tumors from TCGA and COMPASS databases as either epithelial (EPI) or mesenchymal (MES).

Gene signatures were derived from T.Z. Tan et al. EMBO Mol Med. 2014;6(10):1279-93.

**EPI**

| ABHD11 |
| --- |
| AGR2 |
| ARHGAP32 |
| BCAS1 |
| CBLC |
| CEACAM1 |
| CLDN4 |
| CLDN7 |
| ELF3 |
| ELMO3 |
| EPCAM |
| EPS8L1 |
| EPS8L2 |
| ESRP1 |
| ESRP2 |
| EXPH5 |
| F11R |
| FA2H |
| FXYD3 |
| GMDS |
| GRHL2 |
| INAVA |
| JUP |
| KRT18 |
| KRT19 |
| KRT8 |
| LAD1 |
| LLGL2 |
| LSR |
| MLPH |
| MST1R |
| MUC1 |
| NQO1 |
| PKP3 |
| PRSS8 |
| PTK6 |
| RAB25 |
| RAPGEFL1 |
| S100A14 |
| S100P |
| SDC4 |
| SLC44A4 |
| SPAG1 |
| SPDEF |
| SPINT2 |
| ST14 |
| STYK1 |
| TPD52 |
| TRPM4 |
| TUFT1 |

**MES**

| AKT3 |
| --- |
| ANK2 |
| AP1S2 |
| ASPN |
| CALD1 |
| CDK14 |
| CEP170 |
| CHN1 |
| COL15A1 |
| CYP1B1 |
| DDR2 |
| DSE |
| ECM2 |
| EFEMP1 |
| FAP |
| FBN1 |
| FERMT2 |
| FLI1 |
| FSTL1 |
| GIMAP6 |
| GREM1 |
| GUCY1B1 |
| HEG1 |
| IGFBP5 |
| JAM3 |
| KCNJ8 |
| MAFB |
| MAP1B |
| MS4A6A |
| NAP1L3 |
| NUAK1 |
| OLFML3 |
| PLXNC1 |
| RECK |
| RUNX1T1 |
| SACS |
| SDC2 |
| SLIT2 |
| SPARCL1 |
| TNS1 |
| TRPC1 |
| TUBA1A |
| VCAN |
| VIM |
| WIPF1 |
| WWTR1 |
| ZCCHC24 |
| ZEB1 |
| ZEB2 |
| ZFPM2 |
