# Supplementary material for: STAT3 is a genetic modifier of TGF-beta induced EMT in KRAS mutant pancreatic cancer
Source: eLife. 2024 Apr 4;13:RP92559. doi: 10.7554/eLife.92559 (PMC10994661; doi:10.7554/eLife.92559)
Supplement: Figure 4—source data 3. — Singh et al., 2009;15 (Philips et al., 2022):489–500, and genes used to define a KRAS-dependent signature (KRAS_sig) or an RSK-dependent signature (RSK_sig) derived from Yuan et al., 2018;22 (Jerez et al., 2012):1889–902. [file elife-92559-fig4-data3.docx]

**Figure 4 - source data 3:** Genes used to define a RAS Dependency Index (RDI) derived from A.Singh et al. Cancer Cell, 2009;15(6):489-500, and genes used to define a KRAS dependent signature (KRAS_sig) or an RSK dependent signature (RSK_sig) derived from T.L.Yuan et al. Cell Rep. 2018;22(7):1889-902.

| **RDI**  LAMA3 |
| --- |
| TGFA |
| ADAM8 |
| TMEM45B |
| MAOA |
| CEACAM1 |
| S100A14 |
| DAPP1 |
| PRSS22 |
| RDHE2 |
| FAM83A |
| F11R |
| C1orf116 |
| C6orf141 |
| LAD1 |
| IRF6 |
| PRSS8 |
| CDH1 |
| SH2D3A |
| EPS8L1 |

**KRAS_sig**

| MARVELD3 |
| --- |
| CLDN4 |
| DDR1 |
| TPRG1L |
| GJB3 |
| RASSF7 |
| ARRDC1 |
| CCDC64B |
| RAB11FIP1 |
| PKP2 |
| EPS8L1 |
| SOWAHB |
| SSH3 |
| ST14 |
| MACC1 |
| LPAR2 |
| SH3YL1 |
| LSR |
| OSBPL2 |
| TMEM139 |
| F2RL1 |
| PKP3  CBLC |
| **RSK_sig**  MYL6B |
| ATP8B2 |
| TFPI2 |
| NAP1L2 |
| PRKD1 |
| SLC25A4 |
| DENND5A |
| POGLUT2 |
| GNG11 |
| SLC47A1 |
| C1R |
| CRISPLD1 |
| TTC28 |
| SCPEP1 |
| MAGEH1 |
| SLC47A1 |
| SCPEP1 |
| TTC26 |
| OAZ2 |
| ENO1 |
| MRPS7 |
| TMEM160 |
| SMARCD3 |
